# PGK1-coupled HSP90 stabilizes GSK3β expression to regulate the stemness of breast cancer stem cells

**DOI:** 10.20892/j.issn.2095-3941.2020.0362

**Published:** 2021-08-17

**Authors:** Wei Tang, Yu Wu, Xin Qi, Rilei Yu, Zhimin Lu, Ao Chen, Xinglong Fan, Jing Li

**Affiliations:** 1 Key Laboratory of Marine Drugs, Chinese Ministry of Education, School of Medicine and Pharmacy, Ocean University of China, Qingdao 266003, China; 2 Department of Hepatobiliary and Pancreatic Surgery and Zhejiang Provincial Key Laboratory of Pancreatic Disease of the First Affiliated Hospital, Institute of Translational Medicine, Zhejiang University, Hangzhou 310029, China; 3 Department of Thoracic Surgery, Qilu Hospital (Qingdao), Cheeloo College of Medicine, Shandong University, Qingdao 266003, China; 4 Open Studio for Druggability Research of Marine Natural Products, Pilot National Laboratory for Marine Science and Technology (Qingdao), Qingdao 266003, China; 5 Laboratory for Marine Drugs and Bioproducts of Qingdao National Laboratory for Marine Science and Technology, Qingdao 266003, China

**Keywords:** Glycogen synthase kinase-3β (GSK3β), heat shock protein 90 (Hsp90), phosphoglycerate kinase 1 (PGK1), hsp90 inhibitors, breast cancer stem cell

## Abstract

**Objective:**

Glycogen synthase kinase-3β (GSK3β) has been recognized as a suppressor of Wnt/β-catenin signaling, which is critical for the stemness maintenance of breast cancer stem cells. However, the regulatory mechanisms of GSK3β protein expression remain elusive.

**Methods:**

Co-immunoprecipitation and mass spectral assays were performed to identify molecules binding to GSK3β, and to characterize the interactions of GSK3β, heat shock protein 90 (Hsp90), and co-chaperones. The role of PGK1 in Hsp90 chaperoning GSK3β was evaluated by constructing 293T cells stably expressing different domains/mutants of Hsp90α, and by performing a series of binding assays with bacterially purified proteins and clinical specimens. The influences of Hsp90 inhibitors on breast cancer stem cell stemness were investigated by Western blot and mammosphere formation assays.

**Results:**

We showed that GSK3β was a client protein of Hsp90. Hsp90, which did not directly bind to GSK3β, interacted with phosphoglycerate kinase 1 *via* its C-terminal domain, thereby facilitating the binding of GSK3β to Hsp90. GSK3β-bound PGK1 interacted with Hsp90 in the “closed” conformation and stabilized GSK3β expression in an Hsp90 activity-dependent manner. The Hsp90 inhibitor, 17-AAG, rather than HDN-1, disrupted the interaction between Hsp90 and PGK1, and reduced GSK3β expression, resulting in significantly reduced inhibition of β-catenin expression, to maintain the stemness of breast cancer stem cells.

**Conclusions:**

Our findings identified a novel regulatory mechanism of GSK3β expression involving metabolic enzyme PGK1-coupled Hsp90, and highlighted the potential for more effective cancer treatment by selecting Hsp90 inhibitors that do not affect PGK1-regulated GSK3β expression.

## Introduction

Glycogen synthase kinase-3 (GSK-3) was initially found to be a key serine/threonine protein kinase involved in glycogen biosynthesis^1^. GSK-3 has 2 family members, GSK3α and GSK3β, which are ubiquitously expressed and highly conserved^2^. GSK3β is involved in several signal transduction cascades, including the PI3K/Akt/mTOR, Wnt/β-catenin, and MEK/ERK pathways, thereby regulating cell cycle progression, differentiation, survival, embryogenesis, migration, and metabolism^3,4^.

GSK3β has been implicated in a wide range of diseases, including neurodegeneration, inflammation, fibrosis, and cancer^5^. GSK3β suppresses cell growth, the epithelial-mesenchymal transition, and drug-resistance in breast cancer by inhibiting the GSK3β-β-catenin signaling pathway^6,7^, and its inactivation is found in approximately half of invasive mammary carcinomas^8^. GSK3β inactivation stabilizes β-catenin expression, induces its translocation to the nucleus, upregulates the downstream target *CCND1* gene (encoding cyclin D1), and promotes breast cancer stem cell (BCSC) function and mammary tumor development^9,10^. In addition, it has been shown that WAP-Cre-mediated deletion of GSK3β in the mammary epithelium activates Wnt/β-catenin signaling and induces mammary intraepithelial neoplasia^11^.

GSK3β is constitutively active in normal cells and its activity is regulated by multiple posttranslational modifications^12,13^. GSK3β can be suppressed by phosphorylation at Ser (S) 9 by Akt, AGC kinases, MAPK-activated protein kinase 1, p70 ribosomal S6 kinase 1, p90 ribosomal S6 kinase 1, and PKA^12^. In contrast, Src, MEK1/2, Fyn, and Pyk2 induce the activation of GSK3β by phosphorylation at Tyr 216^12^. GSK3β can also be acetylated, which increases its activity^13^.

Heat shock protein 90 (Hsp90) is a molecular chaperone involved in the correct folding, maturation, and stability of multiple proteins (commonly referred to as “client proteins”)^14^. Hsp90 forms a dimer, with monomers that individually consists of an N-terminal ATP-binding domain, a middle domain, and a C-terminal dimerization domain^15^. It has been proposed that different client proteins interact with different interaction sites of Hsp90^16^. Additionally, Hsp90 conformational changes in the N-terminus from an “open” state to a “closed” conformation is essential for its function upon binding and hydrolyzing ATP^17^. Hsp90 function is facilitated by co-chaperones such as HOP/Sti1, CDC37, Aha1, and P23/Sba1, which bind to different domains and conformations of Hsp90 in distinct stages of the Hsp90 chaperone cycle^18^. Hsp90 is probably involved in regulating GSK3β expression and activity, which are decreased in the presence of the Hsp90 N-terminal inhibitor, geldanamycin^19^. However, it has been reported that there was no interaction between wild-type GSK3β and Hsp90, and GSK3β was unaffected by the co-chaperone, CDC37^20,21^. Thus, the mechanism underlying Hsp90-regulated GSK3β expression remains elusive.

In this study, we showed that phosphoglycerate kinase 1 (PGK1) was a newly identified co-chaperone, which was essential for GSK3β binding to Hsp90. In addition, inhibitors targeting different domains of Hsp90 had distinct effects on GSK3β stability by affecting the interaction between Hsp90 and PGK1, resulting in distinct degrees of inhibitory effects on the stemness of BCSCs.

## Materials and methods

### Compounds and reagents

HDN-1 (chetracin B) from the Antarctic fungus, *Oidiodendron truncatum* GW3–13, with purity > 99%, was obtained from the School of Medicine and Pharmacy, Ocean University of China. 17-AAG was purchased from Apollo Scientific (Stockport, UK). EGF, bFGF, and B27 were purchased from Gibco (Rockville, MD, USA). Insulin and FITC-conjugated goat anti-rabbit antibody were purchased from Solarbio (Beijing, China). Rabbit mAb IgG XP isotype control, mouse mAb IgG XP^®^ isotype control, and antibodies to detect ALDH1A1, Hsp70, GSK3β, phospho-GSK3β (ser 9), β-catenin, phospho-β-catenin (ser 45), AKT, phospho-Akt (Ser 473), Erk, HOP, PP5, FKBP5, CDC37, JAK1, Stat3, c-Raf, FGFR, c-Abl, ubiquitin, and Flag were obtained from Cell Signaling Technology (Danvers, MA, USA). Anti-Hsp90 antibody and protein A/G agarose were purchased from Santa Cruz Biotechnology (Dallas, TX, USA). Anti-Aha1 antibody and His-GSK3β protein were obtained from Sino Biological (Beijing, China). Recombinant human Hsp90α protein and anti-P23 antibody were purchased from Abcam (Cambridge, UK). Anti-PGK1 antibody was purchased from Novus Biologicals (Littleton, CO, USA). FITC-conjugated anti-CD44 and APC-conjugated anti-CD24 antibodies were obtained from Thermo Fisher Scientific (Waltham, MA, USA). The primary antibodies (anti-β-actin, anti-tubulin, and anti-glyceraldehyde 3-phosphate dehydrogenase), and the secondary antibodies were purchased from HuaBio (Hangzhou, China). MTT was obtained from Sigma-Aldrich (St. Louis, MO, USA). BeaverBeads™ GSH and BeaverBeads™ IDA-Nickel were purchased from Beaver Biosciences (Guangzhou, China). Phenylmethylsulfonyl fluoride (PMSF), MG132, and 4′,6-diamidino-2-phenylindole (DAPI) were purchased from Beyotime Institute of Biotechnology (Shanghai, China). SiRNA sequences were constructed by GenePharma (Shanghai, China).

### Cell culture

All cell lines were purchased from the Cell Bank of the Chinese Academy of Sciences (Shanghai, China). Adriamycin-resistant MCF-7 (MCF-7ADR) human breast cancer cells and A2780 human ovarian cancer cells were cultured in RPMI-1640 medium (Genom; Hangzhou, China). The 293T cells were cultured in high glucose DMEM. MCF-7 human breast cancer cells and HeLa human cervical cancer cells were maintained in MEM (Genom; Hangzhou, China), with additional 0.01 mg/mL human recombinant insulin for the MCF-7 cells. MDA-MB-231 human breast cancer cells were cultured in L-15 medium (Genom; Hangzhou, China), and A549 human non-small cell lung cancer cells were cultured in F-12K medium (Genom; Hangzhou, China). All media were supplemented with 10% fetal bovine serum (Gibco; Rockville, MD, USA), 100 units/mL penicillin, and 0.1 mg/mL streptomycin. The cells were maintained at 37 °C in a humidified incubator with 5% CO_2_.

### Clinical tissue specimens and lysate preparation

Breast cancer tissues were obtained from Qilu Hospital (Qingdao, China) using the protocol approved by the local ethics committee (Approval No. KYLL-2018012). Informed consent was obtained from all patients. Specimens were surgically removed from breast cancer patients from November, 2020 to December, 2020, and the diagnoses were confirmed by pathological analyses. All pathological and clinical parameters were retrieved from electronic medical records.

Total tissues were homogenized in sample lysis buffer on ice for 40 min. The lysates were incubated with 1 μg of Hsp90 antibody or IgG overnight at 4 °C, and then incubated with protein A/G-agarose for 2 h at 4 °C. The beads were washed 6 times with washing buffer, and resuspended in 2× loading buffer followed by immunoblot analyses.

### MTT assay

The MTT assay was used to measure the inhibitory effect of compounds on the viability of cancer cells. The cells were seeded at a density of 5 × 10^3^ cells/well in 96-well plates. After attachment, the cells were treated with increasing concentrations of compounds. After 48 h, 20 μL of MTT was added to each well of the 96-well plate. After 4 h, the formazan product was dissolved in dimethyl sulfoxide and quantitated spectrophotometrically at a wavelength of 570 nm using a microplate reader (BioTek, Winooski, VT, USA). The IC_50_ value was defined as the concentration that inhibited cell viability by 50%.

### Fluorescence-activated cell sorting (FACS)

To sort CD44^+^CD24^−/low^ and CD44^+^CD24^+^ cells, MCF-7ADR cells were stained with FITC-conjugated anti-CD44 and APC-conjugated anti-CD24 antibodies for 30 min at 4 °C. After washing with cold phosphate-buffered saline (PBS), the cells were resuspended in 0.5 mL of PBS. CD44^+^CD24^−/low^ and CD44^+^CD24^+^ cells were sorted by flow cytometry (MOFLO XDP; Beckman Coulter, Brea, CA, USA).

### Immunofluorescence

CD44^+^CD24^−/low^ cells and CD44^+^CD24^+^ cells were seeded in 384-well microplates with a glass bottom (Corning, NY, USA). After attachment, the cells were fixed with 4% paraformaldehyde in PBS for 30 min and permeabilized with 0.3% Triton X-100 for 15 min at room temperature. After blocking with 1% bovine serum albumin in PBS, the cells were stained overnight with phosphorylated-GSK3β (S9) antibody in blocking buffer at 4 °C, then incubated with secondary FITC-conjugated anti-rabbit IgG antibody for 1 h at room temperature. The resulting images were captured using a laser scanning confocal microscope (Carl Zeiss; Jena, Germany).

### Mammosphere formation assay

Single cell suspensions of MCF-7ADR cells were cultured in 24-well ultra-low attachment plates at a density of 2,000 cells/well. Mammospheres were formed in serum-free DMEM/F12 containing 20 ng/mL EGF, 10 ng/mL bFGF, 2% B27, and 5 μg/mL insulin, and then treated with different concentrations of HDN-1 or 17-AAG for 7 days. The size and number of mammospheres with a diameter > 50 μm per group were evaluated with a Cytation 5 Cell Imaging Multi-Mode Reader (BioTek, Winooski, Vermont, USA).

### Plasmid construction of the Hsp90α domains and mutants

The PcDNA3-PGK1 plasmid with a flag tag was provided by Professor Zhimin Lu from MD Anderson Cancer Center (Houston, TX, USA). Flag-tagged full-length Hsp90α (1–732), N-domain (9–236), M-domain (237–520), and C-domain (535–732) were cloned from human cDNA and inserted into the PLVX-IRES-ZsGreen1 vector (Takara Bio, Kutsatsu, Japan). Flag-tagged Hsp90α E47A and D93A mutants were cloned from the Flag-tagged Hsp90α plasmid followed by incubation with Dpn I (TaKaRa; Tokyo, Japan) at 37 °C for 1.5 h. These constructs were then transformed into DH5α and amplified. The primers for these constructs are shown in **Supplementary Table S1**.

### Transfection with siRNA and DNA plasmids

MCF-7ADR and 293T cells at ˜70% confluency were transfected in 6-well plates with 20 nM siRNA or 2 μg of DNA for 48 h using Lipofectamine 3000 (Invitrogen, Carlsbad, CA, USA) according to the manufacturer’s instructions. The transfection efficiencies were analyzed by Western blot using the indicated antibodies. The siRNA sequences are shown in **Supplementary Table S1**.

### Co-immunoprecipitation (IP) and Western blot analysis

For co-IP, the cells were lysed on ice for 30 min using cell lysis buffer for Western blot and IP (Beyotime Institute of Biotechnology; Shanghai, China) with 1 mM PMSF. After centrifugation at 12,000 × *g* and 4 °C for 15 min, the supernatants were incubated overnight with the indicated antibody or IgG at 4 °C and then incubated for 2 h with protein A/G-agarose beads while rotating at 95 rpm. The beads were washed 6 times with washing buffer (50 mM Tris-HCl, 150 mM NaCl, 1% Triton, pH 7.5), and resuspended in 2× loading buffer (100 mM Tris-HCl, PH 6.8, 4% SDS, 20% Glycerol, 10% β-mercaptoethanol, 0.1% bromophenol blue) followed by immunoblot analysis.

For Western blot analysis, cell lysates were prepared using 2× loading buffer on ice. Equal amounts of protein were separated using SDS-PAGE, and then transferred to nitrocellulose membranes (GE Healthcare; Little Chalfont, Buckinghamshire, UK). The membranes were blotted with primary antibodies followed by secondary antibodies. Proteins on the membranes were detected by chemiluminescence using enhanced chemiluminescence detection reagents (EpiZyme, Shanghai, China).

### Mass spectrometric analysis

MCF-7ADR cells were lysed on ice for 30 min with cell lysis buffer for Western blot and IP with 1 mM PMSF. After centrifugation, GSK3β was immunoprecipitated from the cell lysates using anti-GSK3β antibody or IgG and then incubated for 2 h with protein A/G-agarose beads while rotating at 95 rpm. The beads were washed 6 times with washing buffer, and resuspended in 2× loading buffer. The samples were then analyzed by liquid chromatography-mass spectrometry/mass spectrometry (OE Biotech; Shanghai, China) for the identification of proteins bound with GSK3β.

Candidate GSK3β interacting components were identified using Mascot 2.3 software (Matrix Science, Chicago, IL, USA) and the UniProt-Homo database, on the condition that the number of IgG associated unique peptides = 0, which led to the exclusion of IgG-bound proteins in the GSK3β group.

### Protein expression and purification

His-PGK1 plasmid was provided by Professor Zhimin Lu from MD Anderson Cancer Center. Plasmids encoding the GST-C/M-domain of Hsp90α were constructed in our laboratory^22^.

His-PGK1 protein expression was induced by 0.5 mM isopropyl β- d-1-thiogalactopyranoside (IPTG) overnight at 180 rpm and 18 °C, and affinity purified by BeaverBeads™ IDA-Nickel (Beaver Biomedical Engineering, Suzhou, China) in lysis buffer containing 20 mM Na_3_PO_4_, 500 mM NaCl, 5 mM imidazole, 5% glycerol, and 1 mM PMSF at 4 °C for 3 h. After incubation, the beads were washed twice using washing buffer containing 20 mM Na_3_PO_4_, 500 mM NaCl, 50 mM imidazole, 5% glycerol, and 1 mM PMSF. Protein was then eluted using buffer containing 20 mM Na_3_PO_4_, 500 mM NaCl, 500 mM imidazole, 5% glycerol, and 1 mM PMSF. The proteins were > 90% pure, as determined by SDS-PAGE. Concentrations were determined using a BCA assay, and the protein aliquots were stored at −80 °C.

The GST-C/M-domains of Hsp90α proteins were induced by 0.5 mM IPTG treatment overnight with rotation at 180 rpm and 18 °C, then affinity purified using BeaverBeads™ GSH in the lysis buffer containing 20 mM Tris-HCl (pH 8.0), 100 mM KCl, 10% glycerol, 1 mM dithiothreitol (DTT), 0.5% Triton X-100, and 0.5 mM PMSF at 4 °C for 3 h. After incubation, the beads were washed twice using washing buffer containing 20 mM Tris-HCl (pH 8.0), 100 mM KCl, 10% glycerol, 1 mM DTT, and 0.5 mM PMSF. Proteins were then eluted using buffer containing 100 mM Tris-HCl (pH 8.0), 100 mM KCl, 0.1% 2-mercaptoethanol, 2 mM GSH, and 0.5 mM PMSF. The proteins were > 90% pure as determined by SDS-PAGE. Concentrations were determined using BCA assays, and protein aliquots were stored at −80 °C.

### *In vitro* binding assay

The binding of PGK1 with GSK3β or/and different domains (WT/C/M) of Hsp90α was measured by combining 2.5 μg PGK1 with 2.5 μg GSK3β or/and 5 μg of different Hsp90α domains in 300 μL of binding buffer containing 10 mM Tris-HCl, pH 7.5, 100 mM KCl, 2 mM DTT, 0.01% Nonidet P-40, with or without ATP^23^. Then, 5 μg of different Hsp90 domains and 2.5 μg His-PGK1 were incubated for 1 h at 30 °C, and 2.5 μg His-GSK3β was added at 30 °C and incubated for 1 h. To determine the effects of HDN-1 and 17-AAG on the formation of the Hsp90-PGK1-GSK3β complex, excess compounds were added, and the mixture was treated with 1 μM HDN-1 or 17-AAG at 4 °C for 1 h after incubation. After chilling on ice for 30 min, the samples were incubated for 1 h with anti-Hsp90 antibody at 4 °C, and bound to protein A/G-Sepharose for 2 h at 4 °C. The resin pellets were washed 5 times with 700 μL of binding buffer and subjected to Western blot.

### Molecular docking

Docking of PGK1 to Hsp90 was conducted using the HDOCK webserver (http://hdock.phys.hust.edu.cn/)^24^. There was a relatively high conservation within these 2 isoforms, including Hsp90α and Hsp90β (85% sequence identity)^25^. Because the crystal structure of Hsp90α in the “closed” state was not available, the crystal structure of the “closed” conformation of Hsp90β [protein database (PDB) code: 5FWK] was used as a replacement of Hsp90α in the “closed” state for docking. The crystal structures of Hsp90β (PDB code: 5FWK) and PGK1 (PDB code: 1VJD) were retrieved from the PDB, and submitted to the HDDOCK webserver. The results were analyzed using PyMOL (https://pymol.org/2/).

### Statistical analysis

The data in the graphs are presented as the mean ± SEM. Statistical comparisons among groups were conducted using Student’s *t*-test for at least 3 independent experiments. Asterisks in figures indicate significant differences (*P < 0.05; **P < 0.01).

## Results

### GSK3β is a client protein of Hsp90

**Figure 1:**
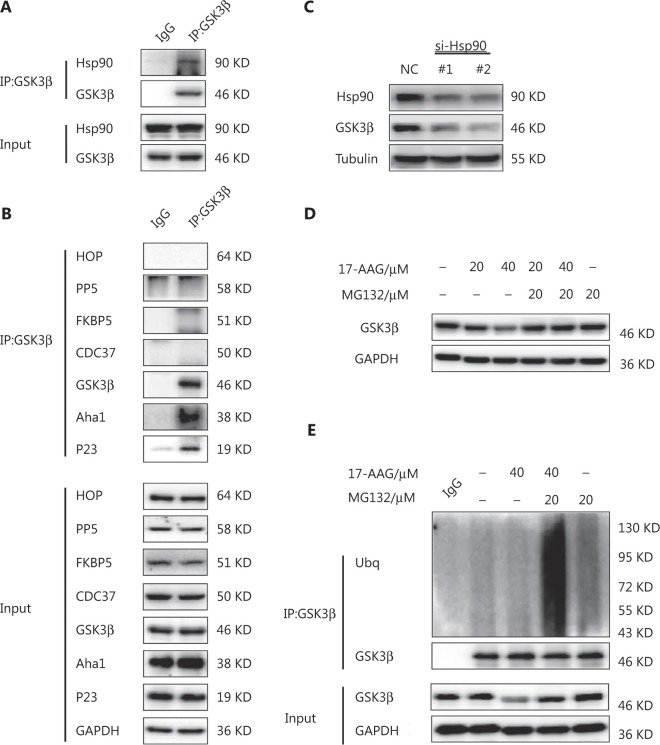
Glycogen synthase kinase-3 (GSK3β) is a client protein of heat shock protein 90 (Hsp90). (A) The interaction between GSK3β and Hsp90 in MCF-7ADR cells detected by a co-immunoprecipitation (IP) assay. GSK3β was immunoprecipitated from cell lysates using an anti-GSK3β antibody. Immunoblotting analyses were performed with the indicated antibodies. (B) The interaction of GSK3β with Hsp90 co-chaperones in MCF-7ADR cells using a co-IP assay. GSK3β was immunoprecipitated from cell lysates using an anti-GSK3β antibody. Immunoblotting analyses were performed with the indicated antibodies. (C) The effect of Hsp90 knockdown on GSK3β expression. MCF-7ADR cells were transfected with a control siRNA (NC) or Hsp90 siRNA for 48 h. Immunoblotting analyses were performed with the indicated antibodies. (D) The effect of 17-AAG on GSK3β expression in MCF-7ADR cells. The cells were treated with 17-AAG for 12 h, followed by co-treatment with MG132 for 12 h. Immunoblotting analyses were performed with the indicated antibodies. (E) The effect of 17-AAG on polyubiquitination of GSK3β. MCF-7ADR cells were treated with 17-AAG for 12 h, followed by co-treatment with MG132 for 12 h. GSK3β was immunoprecipitated from cell lysates using an anti-GSK3β antibody Immunoblotting analyses were performed with the indicated antibodies. All experiments were performed independently and repeated at least 3 times.

To determine the mechanism responsible for the Hsp90 regulation of GSK3β, we performed a co-IP assay and confirmed the interaction between GSK3β and Hsp90 (**Figure 1A**). IP with an anti-GSK3β antibody showed that Aha1 and P23, but not HOP, PP5, FKBP5, or CDC37, interacted with GSK3β (**Figure 1B**). In addition, immunoblotting showed that Hsp90 depletion significantly reduced GSK3β expression in MCF-7ADR human breast cancer cells (**Figure 1C**). Together, these results supported the hypothesis that GSK3β was a client protein of Hsp90.

Hsp90 inhibitors induce degradation of Hsp90 clients by the ubiquitin-proteasome pathway^18^. By targeting N-terminal ATP/ADP-binding domain of Hsp90, 17-AAG has been shown to be a specific Hsp90 inhibitor^26^. Treatment with 17-AAG reduced GSK3β expression in MCF-7ADR cells, which was alleviated by co-treatment with the MG132 proteasome inhibitor (**Figure 1D**). Consistently, GSK3β polyubiquitination was enhanced by co-treatment with the Hsp90 N-terminal inhibitor, 17-AAG and MG132 (**Figure 1E**). These results indicated that GSK3β was degraded *via* the ubiquitin-proteasome pathway mediated by the Hsp90 inhibitor, further supporting the idea that Hsp90 regulated GSK3β levels.

### PGK1 acts as an Hsp90 co-chaperone that specifically regulates GSK3β expression

**Figure 2:**
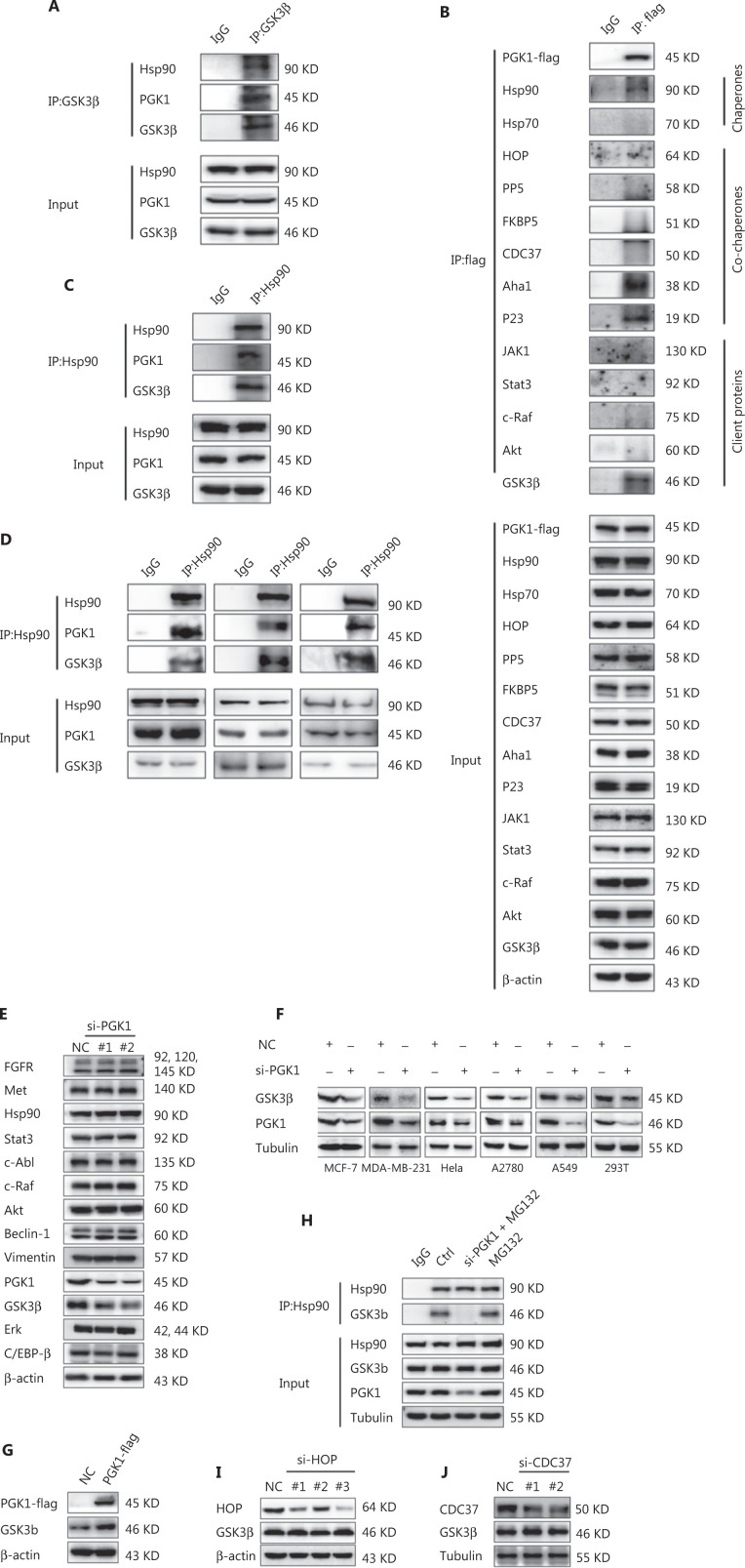
PGK1 acts as an Hsp90 co-chaperone specifically regulating glycogen synthase kinase-3 (GSK3β) expression. (A) The interaction of GSK3β with Hsp90 and PGK1 in MCF-7ADR cells. GSK3β was immunoprecipitated using an anti-GSK3β antibody. Immunoblotting analyses were performed with the indicated antibodies. (B) The interaction of PGK1 with chaperones, co-chaperones, and client proteins. MCF-7ADR cells were transfected with 2 μg PGK1-Flag plasmid for 48 h. PGK1-Flag was immunoprecipitated using an anti-flag antibody. Immunoblotting analyses were performed with the indicated antibodies. (C) The interaction of Hsp90 with GSK3β and PGK1 in MCF-7ADR cells. Hsp90 was immunoprecipitated from cell lysates using an anti-Hsp90 antibody. Immunoblotting analyses were performed with the indicated antibodies. (D) The interaction of Hsp90 with GSK3β and PGK1 in clinical breast cancer tissues of 3 patients. Hsp90 was immunoprecipitated from lysates using an anti-Hsp90 antibody. Immunoblotting analyses were performed with the indicated antibodies. (E, G) PGK1 depletion (E) or (G) overexpression on client protein expression of Hsp90. MCF-7ADR cells were transfected with PGK1 siRNA (E) or PGK1-Flag (G) for 48 h. Immunoblotting analyses were performed with the indicated antibodies. (F) The effect of PGK1 knockdown on GSK3β expression in the indicated cells. Immunoblotting analyses were performed with the indicated antibodies. (H) The effect of PGK1 knockdown on the interaction between Hsp90 and GSK3β. MCF-7ADR cells were transfected with PGK1 siRNA. After 36 h, cells were treated with 20 μM MG132 for 12 h, and then subjected to IP using an anti-Hsp90 antibody. Immunoblotting analyses were performed with the indicated antibodies. (I, J) The effect of HOP (I) or CDC37 (J) knockdown on GSK3β expression. MCF-7ADR cells were transfected with siRNA for 48 h. Immunoblotting analyses were performed with the indicated antibodies. All experiments were performed independently and repeated at least 3 times.

As co-chaperones, HOP, an adaptor that binds to the C-terminus of Hsp90, transfers client proteins from Hsp70-Hsp40 to Hsp90, and CDC37 directs client proteins into the Hsp90 chaperone cycle by binding to the N-terminus of Hsp90^15,27^. Because GSK3β did not interact with HOP or CDC37 (**Figure 1B**), we wondered whether an unknown Hsp90 co-chaperone was involved in GSK3β stability. Mass spectrometry analyses of GSK3β immunoprecipitates from MCF-7ADR cells identified 117 GSK3β interacting proteins (**Supplementary Table S2**). Among these candidates, 21 proteins had scores higher than 90 (**Table 1**). Phosphoglycerate kinase 1 (PGK1, score = 99) has been documented as enhancing Hsp90 chaperone activity by its interaction with Hsp90^28^, thereby exhibiting non-metabolic functions during tumorigenesis^29–32^. There has been no such report for other proteins with high scores. This interaction was validated by reciprocal IP assays with anti-GSK3β (**Figure 2A**) or anti-Flag-tagged PGK1 antibodies (**Figure 2B**). In addition, Hsp90, Aha1, and P23, but not HOP, CDC37, PP5, or FKBP5, were detected in PGK1 immunoprecipitates (**Figure 2B**). The interaction between PGK1 and Hsp90 was also detected by IP analysis with an anti-Hsp90 antibody (**Figure 2C**). Together, these results indicated that Hsp90, GSK3β, and PGK1 interacted with each other. The interaction of the 3 molecules was further validated in clinical breast cancer tissues (**Figure 2D**).

**Table 1:** Mass spectrometry analysis of proteins (scores > 90) interacting with glycogen synthase kinase-3

Accession	Score*	Mass	Matches^†^	Sequences^‡^	emPAI^§^
sp|P35579|MYH9_HUMAN	1,026	227,646	47	32	0.84
sp|P60709|ACTB_HUMAN	344	42,052	18	10	2.11
tr|A0A075B6Z2|A0A075B6Z2_HUMAN	248	2,220	27	1	1.81
tr|H0YH81|H0YH81_HUMAN	189	38,226	7	5	0.65
sp|P06702|S10A9_HUMAN	159	13,291	7	2	1.49
sp|Q02413|DSG1_HUMAN	139	114,702	7	7	0.22
sp|P31944|CASPE_HUMAN	138	27,947	5	5	0.76
sp|P81605|DCD_HUMAN	133	11,391	5	5	2.73
tr|A0A0C4DGB6|A0A0C4DGB6_HUMAN	121	71,177	4	4	0.2
tr|A0A087WVQ9|A0A087WVQ9_HUMAN	116	48,195	5	4	0.39
sp|Q6UWP8|SBSN_HUMAN	114	60,562	6	4	0.3
sp|P01040|CYTA_HUMAN	113	11,000	4	4	1.98
sp|P14923|PLAK_HUMAN	109	82,434	5	4	0.22
**sp|P00558|PGK1_HUMAN**	**99** ^※^	**44,985** ^※^	**5** ^※^	**3** ^※^	**0.43** ^※^
sp|P15924|DESP_HUMAN	96	334,021	6	4	0.05
tr|F8VPF3|F8VPF3_HUMAN	95	14,598	3	2	0.87
sp|P12273|PIP_HUMAN	94	16,847	3	3	0.73
sp|Q9ULV4|COR1C_HUMAN	92	53,899	5	4	0.27
sp|P29508|SPB3_HUMAN	91	44,594	7	5	0.43
sp|P31151|S10A7_HUMAN	90	11,578	5	5	2.69
sp|P68871|HBB_HUMAN	90	16,102	3	3	0.77

*Score: Protein scores, calculated using Proteome Discoverer from a list of peptides identified for a particular protein, indicated the relevance of the protein. ^†^Matches: the number of high confidence images of mass spectrometry, which matched the protein (confidence interval > 95%). ^‡^Sequences: the number of high confidence peptides, which matched the protein. ^§^emPAI: the content of proteins. ^※^Bold values are parameters of PGK1 in mass spectrometry analysis.

To determine whether PGK1 regulated GSK3β expression, we depleted PGK1 in MCF-7ADR cells and found that PGK1 knockdown significantly reduced GSK3β expression (**Figure 2E**). A similar reduction was also observed after PGK1 depletion in 293T renal epithelial cells, MCF-7 and MDA-MB-231 breast cancer cells, HeLa cervical cancer cells, A2780 ovarian cancer cells, and A549 non-small cell lung cancer cells (**Figure 2F**). Conversely, PGK1 overexpression augmented GSK3β expression (**Figure 2G**). Notably, PGK1 depletion significantly inhibited the binding of Hsp90 to GSK3β (**Figure 2H**), suggesting that PGK1 was a co-chaperone of Hsp90, which facilitated Hsp90 binding to GSK3β. Consistent with the lack of association of PGK1 with some Hsp90 co-chaperones, such as HOP, CDC37, PP5, or FKBP5 (**Figure 2B**), PGK1 depletion did not affect the expression of other Hsp90 client proteins, including FGFR, Met, Stat3, c-Abl, c-Raf, Akt, Beclin-1, Vimentin, Erk, and C/EBP-β (**Figure 2E**), a finding confirmed by the observation that some of these client proteins did not interact with PGK1 (**Figure 2B**). In contrast, HOP or CDC37 depletion, which reduced the expressions of Met, AKT and c-Raf^33–35^, had no effect on the protein levels of GSK3β (**Figure 2I and 2J**). Taken together, these results suggested that PGK1 acted as an Hsp90 co-chaperone to specifically regulate GSK3β expression.

### PGK1 binds to the C-terminus of Hsp90 in the “closed” state to promote interaction between GSK3β and Hsp90

**Figure 3:**
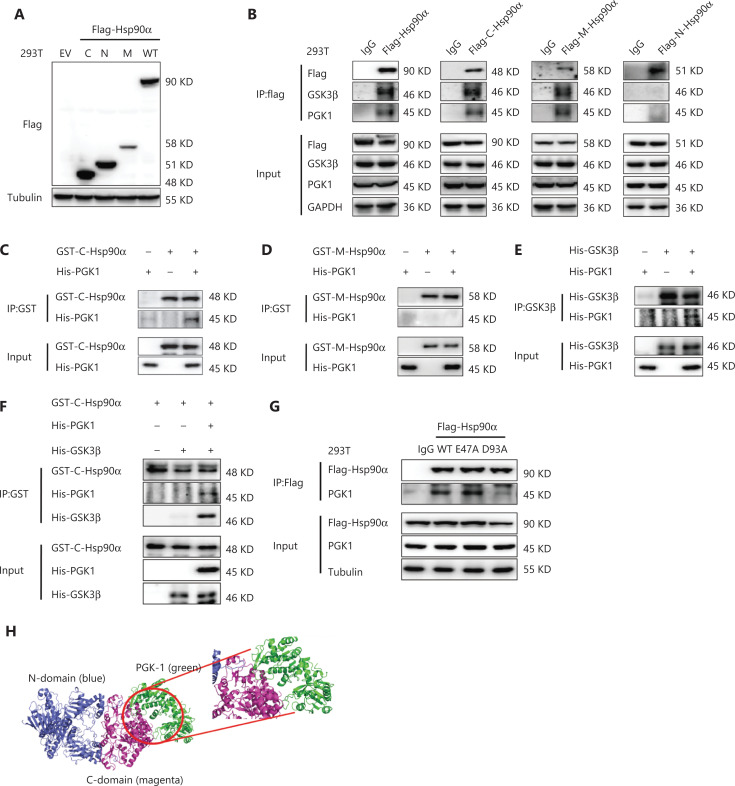
PGK1 binds to the C-terminus of Hsp90 in the “closed” state to promote the interaction between glycogen synthase kinase-3 GSK3β and Hsp90. (A) Constructed 293T cells stably expressing flag-tagged Hsp90α full-length (WT), N-, M-, or C- domains. Empty vector was used as a control. (B) The interaction of different Hsp90α domains with PGK1 and GSK3β. Different domains of Flag-Hsp90α were immunoprecipitated using an anti-Flag antibody. Immunoblotting analyses were performed with the indicated antibodies. (C, D) The interaction of PGK1 with the C-terminus (C) or M-domain (D) of Hsp90α *in vitro*. A total of 5 μg GST-C/M-Hsp90α and 2.5 μg His-PGK1 were incubated at 30 °C for 1 h. Immunoprecipitation (IP) was performed with an anti-GST antibody. Immunoblotting analyses were performed with the indicated antibodies. (E) The interaction of PGK1 with GSK3β *in vitro*. A total of 2.5 μg His-GSK3β and 2.5 μg His-PGK1 were incubated at 30 °C for 1 h. IP was performed with an anti-GSK3β antibody. Immunoblotting analyses were performed with the indicated antibodies. (F) PGK1 regulates the interaction between C-terminal Hsp90α and GSK3β *in vitro*. A total of 5 μg GST-C-Hsp90α and 2.5 μg His-GSK3β were incubated with or without 2.5 μg His-PGK1. IP was performed with an anti-GST antibody. Immunoblotting analyses were performed with the indicated antibodies. (G) The interaction of PGK1 with different conformations of Hsp90α. Flag-tagged Hsp90α wild type, D93A, or E47A mutant was stably expressed in 293T cells. Flag-tagged Hsp90 were immunoprecipitated. Immunoblotting analyses were performed with the indicated antibodies. (H) The binding mode of PGK1 with the “closed” conformation of Hsp90β. The N-terminal and C-terminal of Hsp90β are colored in blue and magenta, respectively, while the PGK1 is shown in green. All experiments were performed independently and repeated at least 3 times.

Hsp90 contains N-, M-, and C-domains for binding different client proteins^16^. We constructed 293T cells stably expressing Flag-tagged full-length Hsp90α, C-, N-, or M-domains (**Figure 3A**) followed by co-IP assays, and showed that PGK1 and GSK3β interacted with the C-domain and M-domain of Hsp90 (**Figure 3B**). Given that co-IP can reflect both direct and indirect interactions between proteins, we mixed bacterially purified His-PGK1 with GST-tagged C- or M-domains of Hsp90α. A GST pulldown assay showed that PGK1 directly interacted with the C-domain (**Figure 3C**), but not the M-domain of Hsp90α (**Figure 3D**), indicating that PGK1 directly bound to the C-domain of Hsp90α.

In addition, incubation of bacterially purified His-PGK1 with bacterially-purified His-GSK3β showed that these 2 proteins directly bound to each other (**Figure 3E**). In contrast, purified GSK3β failed to associate with the purified C-terminus of Hsp90α; however, inclusion of purified PGK1 enabled the interaction between the C-terminal Hsp90α and GSK3β (**Figure 3F**). Together, these results suggested that PGK1 acted as an adapter to facilitate GSK3β binding to Hsp90.

Dynamic conformational alteration of Hsp90 between the open state and the closed state is essential for its function in maturation and stability of client proteins, and co-chaperones interact with Hsp90 in specific conformational states^15^. It has been reported that E47A and D93A mutants of Hsp90α induce the “closed” and “open” conformations, respectively^36^. We showed that expressions of Hsp90α D93A, but not Hsp90α E47A, reduced the binding of Hsp90α to PGK1 in 293T cells (**Figure 3G**), suggesting that Hsp90α in the “closed” conformation bound to PGK1. Molecular docking revealed that PGK1 bound to the C-domain of “closed” Hsp90β by forming extensive pairwise interactions at their binding interface (**Figure 3H**). Together, these results indicated that PGK1 acted as an Hsp90 co-chaperone and bound to the C-terminus of Hsp90 in the “closed” state.

### Hsp90 inhibitors targeting different domains of Hsp90 exhibit distinct degrees of inhibitory effects on BCSC stemness

**Figure 4:**
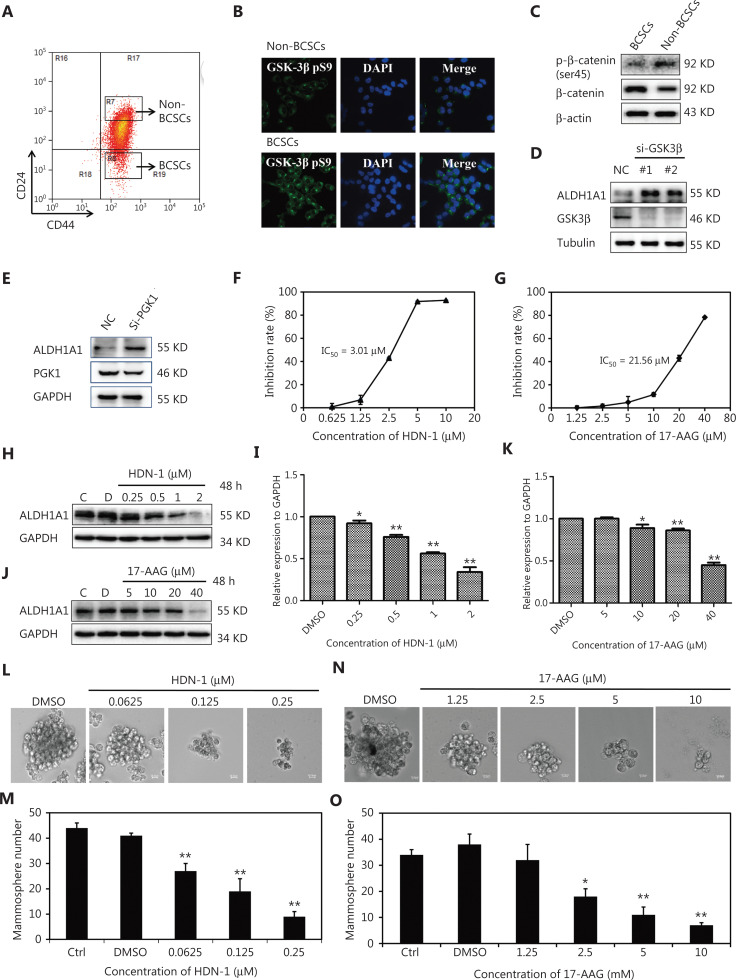
Hsp90 inhibitors targeting different domains of Hsp90 exhibit distinct inhibitory effects on BCSCs. (A) Flow cytometry (FCM) sorting of CD44^+^CD24^−/low^ cells (BCSCs) and CD44^+^CD24^+^ cells (non-BCSCs) from MCF-7ADR cells. (B) Immunofluorescence analysis of BCSCs and non-BCSCs. CD44^+^CD24^−/low^ cells and CD44^+^CD24^+^ cells were stained with an anti-GSK3β pS9 antibody and 4′,6-diamidino-2-phenylindole. (C) Immunoblotting analysis of BCSCs and non-BCSCs were performed with the indicated antibodies. (D) The effect of glycogen synthase kinase-3 knockdown on ALDH1A1 expression. MCF-7ADR cells were transfected with siRNA for 48 h. Immunoblot analyses were performed with the indicated antibodies. (E) The effect of PGK1 knockdown on ALDH1A1 expression. MCF-7ADR cells were transfected with siRNA for 48 h. Immunoblot analyses were performed with the indicated antibodies. (F, G) The effects of HDN-1 (F) and 17-AAG (G) on the proliferation of MCF-7ADR cells. The cells were treated with HDN-1 or 17-AAG for 48 h. Cell proliferation was determined using the MTT assay. (H-K) The effects of HDN-1 (H, I) and 17-AAG (J, K) on ALDH1A1 expression in MCF-7ADR cells. Cells were treated with HDN-1 or 17-AAG for 48 h. Immunoblot analyses of BCSCs and non-BCSCs were performed with the indicated antibodies. (L-O) The effects of HDN-1 (L, M) and 17-AAG (N, O) on mammosphere formation of MCF-7ADR cells. A total of 2,000 MCF-7ADR cells per well were cultured in serum-free medium and then treated with different concentrations of HDN-1 or 17-AAG. After 7 days, images were captured using a light microscope with a camera, and the number of mammospheres was quantified. The scale bar is 10.0 μm. *P < 0.05; **P < 0.01 *vs*. dimethyl sulfoxide. All experiments were performed independently and repeated at least 3 times.

S9 phosphorylation of GSK3β inhibits its activity, resulting in the stabilization of β-catenin by decreasing S45 phosphorylation^12^. Aberrant Wnt/β-catenin signaling in many human malignancies including breast cancer regulates cancer cell survival, proliferation, invasion, and stemness of cancer stem cells (CSCs)^37^. We sorted CD44^+^CD24^−/low^ cells (BCSCs) and CD44^+^CD24^+^ cells (non-BCSCs) from MCF-7ADR breast cancer cells by fluorescence-activated cell sorting (**Figure 4A**). Immunofluorescent staining showed that GSK3β S9 phosphorylation levels were much higher in BCSCs than in non-BCSCs (**Figure 4B**). Correspondingly, decreased β-catenin S45 phosphorylation and increased protein expression were detected in BCSCs, when compared to these levels in non-BCSCs (**Figure 4C**). ALDH1A1 is overexpressed in CSCs and recognized as a marker for CSCs^38^. As expected, we found that GSK3β or PGK1 depletion using their siRNAs significantly induced ALDH1A1 expression (**Figure 4D and 4E**), suggesting that both GSK3β and PGK1 negatively modulated the stemness of breast cancer cells.

To determine whether Hsp90 regulated BCSC stemness through GSK3β, we treated MCF-7ADR cells with the Hsp90 C-terminal inhibitor, HDN-1^26^, and the N-terminal inhibitor, 17-AAG^39^. HDN-1, an epipolythiopiperazine-2,5-dione (ETP) compound obtained from the Antarctic fungus, *Oidiodendron truncatum* GW3–13, which has been identified as a newly discovered Hsp90 C-terminal inhibitor^39^. MCF-7ADR cell proliferation was significantly inhibited by HDN-1 with a low IC_50_ (IC_50_: 3.01 μM) (**Figure 4F**) and 17-AAG with a high IC_50_ (IC_50_: 21.56 μM) (**Figure 4G**). In addition, HDN-1 significantly inhibited ALDH1A1 expression with an IC_50_ of 1.20 μM (**Figure 4H and 4I**), which was much lower than that of 17-AAG (IC_50_: 31.01 μM) (**Figure 4J and 4K**), indicating that HDN-1 had a stronger inhibitory effect than 17-AAG on MCF-7ADR proliferation and ALDH1A1 expression. Consistently, mammosphere formation assays, widely used in evaluating the stemness of BCSCs^40^, showed that HDN-1 significantly inhibited mammosphere formation of MCF-7ADR cells with an IC_50_ of 0.095 μM (**Figure 4L and 4M**), which was approximately 32-fold stronger than its effect on whole cell proliferation (**Figure 4E**). In contrast, 17-AAG had a much higher IC_50_ (4.28 μM) (**Figure 4N and 4O**), and its inhibition of mammosphere formation was only 5-fold stronger than that on whole cell proliferation. These results demonstrated that Hsp90 inhibitors targeting different domains of Hsp90 exhibited distinct degrees of inhibitory effects on BCSC stemness.

### 17-AAG and HDN-1 have distinct effects on Hsp90-PGK1 interaction and subsequent expression of GSK3β and β-catenin

**Figure 5:**
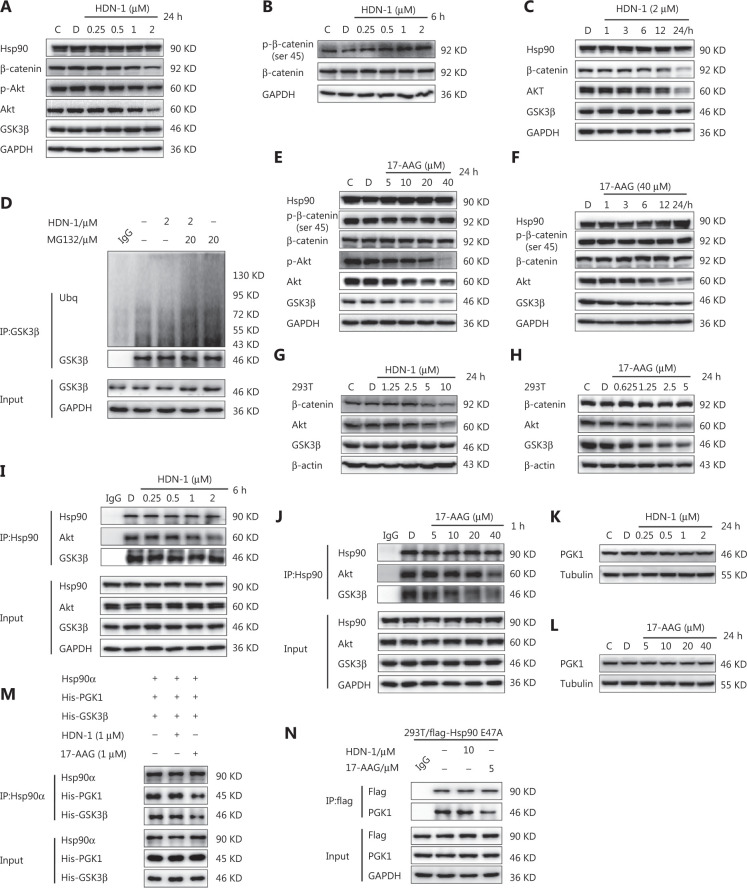
17-AAG and HDN-1 have distinct effects on Hsp90-PGK1 interaction and subsequent expressions of GSK3β and β-catenin. (A, C) The effect of HDN-1 in a dose-dependent (A) or time-course (C) treatment on the expressions of proteins related to Akt-GSK3β-β-catenin signaling in MCF-7ADR cells for 24 h. (B) The effect of HDN-1 on the phosphorylation level of β-catenin at Ser 45 in MCF-7ADR cells. (D) The effect of HDN-1 on polyubiquitination of glycogen synthase kinase-3 (GSK3β). MCF-7ADR cells were treated with HDN-1 for 12 h, followed by co-treatment with MG132 for 12 h. GSK3β was immunoprecipitated from cell lysates using an anti-GSK3β antibody. (E, F) The effect of 17-AAG in a dose-dependent (E) or time-course (F) treatment on the expression of Akt, GSK3β, p-β-catenin at Ser45, and β-catenin in MCF-7ADR cells. (G, H) The effects of HDN-1 (G) and 17-AAG (H) on the expressions of Akt, GSK3β, and β-catenin. The 293T cells were treated with HDN-1 or 17-AAG for 24 h. (I, J) The effects of HDN-1 (I) and 17-AAG (J) on the binding of Hsp90 to Akt and GSK3β. MCF-7ADR cells were treated with HDN-1 or 17-AAG. Hsp90 was immunoprecipitated from cell lysates using an anti-Hsp90 antibody. (K, L) The effects of HDN-1 (K) and 17-AAG (L) on PGK1 expression. (M) The effects of HDN-1 and 17-AAG on the formation of the Hsp90α-PGK1-GSK3β complex *in vitro*. Hsp90α, His-PGK1, and His-GSK3β were incubated, followed by treatment with 1 μM HDN-1 or 17-AAG at 4 °C for 1 h. Immunoprecipitation was performed using an anti-Hsp90 antibody. (N) The effects of HDN-1 and 17-AAG on the interaction between PGK1 and the “closed” conformational Hsp90α. The 293T cells expressing Flag-tagged Hsp90-E47A were treated with HDN-1 or 17-AAG for 24 h. Flag-Hsp90-E47A was then immunoprecipitated from cell lysates. Immunoblotting analyses were performed with the indicated antibodies. All experiments were performed independently and repeated at least 3 times.

We next determined the mechanism responsible for the distinct effect of 17-AAG and HDN-1 on BCSC stemness. AKT phosphorylates GSK3β S9 and inhibits GSK3β stability^12^. HDN-1 inhibited AKT expression and phosphorylation (**Figure 5A**) with correspondingly increased GSK3β-mediated β-catenin S45 phosphorylation (**Figure 5B**) and degradation in a dose- and time-dependent manner (**Figure 5A and 5C**). Notably, HDN-1 did not affect GSK3β polyubiquitination or expression (**Figure 5D**). In contrast, 17-AAG reduced both AKT and GSK3β expression in a dose- (**Figure 5E**) and time- (**Figure 5F**) dependent manner in MCF-7ADR cells; however it did not affect β-catenin phosphorylation and expression (**Figure 5E and 5F**), likely because GSK3β-dependent β-catenin phosphorylation was neutralized by reduced GSK3β expression and reduced AKT-mediated GSK3β phosphorylation and inhibition. Distinct effects of HDN-1 and 17-AAG on the expressions of AKT, GSK3β, and β-catenin were also observed in 293T cells (**Figure 5G and 5H**). These results indicated that HDN-1 and 17-AAG exerted distinct effects on the expressions of AKT-GSK3β-β-catenin cascade proteins.

AKT is known as a client protein of Hsp90^41^. To determine whether the distinct effects of HDN-1 and 17-AAG on β-catenin expression resulted from distinct inhibition of the interactions of AKT and GSK3β with Hsp90, we conducted co-IP assays and showed that HDN-1 treatment reduced the interaction between Hsp90 and AKT in a dose-dependent manner, without affecting the binding of Hsp90 to GSK3β (**Figure 5I**). In contrast, 17-AAG decreased the binding of Hsp90 to both AKT and GSK3β (**Figure 5J**). These results indicated that 17-AAG, but not HDN-1, decreased GSK3β stability by decreasing the interaction between Hsp90 and GSK3β.

**Figure 6:**
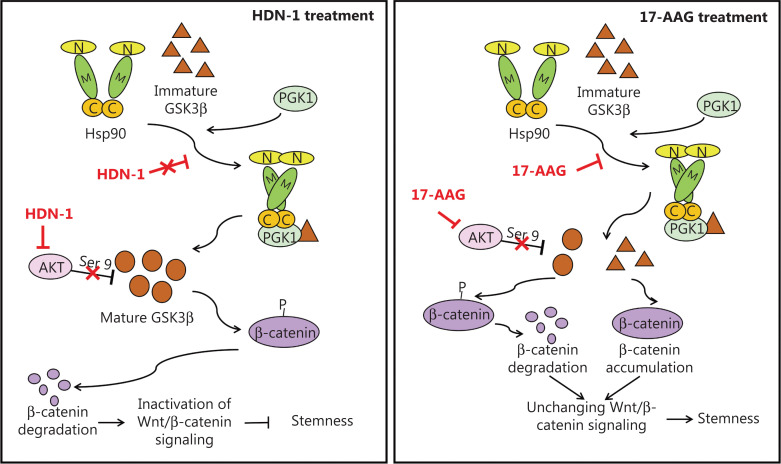
Schematic of the distinct effects of HND-1 and 17-AAG on stemness of breast cancer by affecting Hsp90 regulation of glycogen synthase kinase-3 (GSK3β). The 17-AAG and HDN-1 had distinct effects on Hsp90-PGK1 interaction, resulting in different changes and the instability of GSK3β. They all inhibited AKT expression and its phosphorylation. HDN-1 significantly inhibited the Wnt-β-catenin cascade, yet 17-AAG did not affect the signaling pathway, leading to different effects on the stemness of breast cancer.

17-AAG suppresses Hsp90 N-terminus-dependent Hsp90 dimerization and its conformation in a close state^42^. Consistent with the finding that PGK1 binds to Hsp90 in its closed state, an *in vitro* binding assay showed that 17-AAG, but not HDN-1, disrupted the Hsp90α-PGK1-GSK3β complex (**Figure 5M**), although neither inhibitor affected PGK1 expression (**Figure 5K and 5L)**. In addition, 17-AAG, but not HDN-1, effectively inhibited PGK1 binding to Hsp90 E47A, a closed state mutant, in 293T cells (**Figure 5N**). These results indicated that 17-AAG and HDN-1 had distinct effects on Hsp90-PGK1 interaction and subsequent expression of GSK3β and β-catenin. They also implied that 17-AAG was less effective at inhibiting breast cancer cell stemness than HDN-1 because 17-AAG did not inhibit the AKT-GSK3β-β-catenin cascade (**Figure 6**).

## Discussion

Growing evidence suggests that GSK3β is necessary for the progression of many diseases, including neurodegenerative diseases, type 2 diabetes, and cancers (e.g., glioblastoma, breast cancer, and melanoma)^5^. Previous studies revealed that the role of GSK3β in cancer progression were context-dependent. In some cancers, such as human pancreatic carcinoma, GSK3β overexpression has been positively associated with tumorigenesis and cancer development^43^. In contrast, GSK3β functions as a tumor suppressor in other cancers such as breast cancer^7^. Increasing evidence has shown that CSCs play a critical role in tumorigenesis, development, recurrence, drug-resistance, and eventual metastasis of breast cancer^44^. The malignant properties of CSCs, including self-renewal, differentiation, and chemoresistance, are abnormally regulated by several signaling pathways, such as the Wnt/β-catenin pathway^44^. GSK3β acts as a suppressor of β-catenin nuclear translocation in the Wnt/β-catenin signaling pathway, which is highly activated in CSCs of various cancer types, such as breast cancer^45^. In the present study, our findings showed that GSK3β was a client protein of Hsp90. Hsp90 stabilized GSK3β mediated by PGK1, a newly discovered co-chaperone, and inhibitors targeting different domains of Hsp90 had different effects on GSK3β stability by affecting the interaction of Hsp90-PGK1, resulting in distinct degrees of inhibitory effects on BCSC stemness.

We found that co-chaperones, including Aha1 and P23, bound to GSK3β, but other co-chaperones such as FKBP5, PP5, HOP, and CDC37 did not bind to GSK3β. Aha1 promotes conformational changes leading to the formation of the closed state of Hsp90 by stimulating ATPase activity of Hsp90^46^; P23/Sba1 is involved in the stabilization of the “closed” conformation of Hsp90^15^. Moreover, we found that PGK1 also interacted with Aha1 and P23. These results are consistent with our additional findings that PGK1 recruited GSK3β to bind to Hsp90 in the closed conformation. The results confirmed that some clients interacted strongly with HSP90 in an ATP-stabilized conformation^47^.

HOP and CDC37 are intensively studied recruiter co-chaperones^17^. HOP transfers client proteins to Hsp90 by interacting with the MEEVD motif of the “open” conformation of Hsp90^17^; CDC37 acts in the early chaperone cycle and stabilizes the open conformation of Hsp90 by preventing lid closure and by associating with the N-domain of Hsp90^17^. Our studies demonstrated that HOP and CDC37 were not the co-chaperones of GSK3β^21^. We found that Hsp90, GSK3β, and PGK1 interacted with each other, and that PGK1 bound to the C-domain of Hsp90. PGK1 regulated GSK3β expression without affecting other Hsp90 clients. Furthermore, PGK1 knockdown significantly inhibited the interaction between Hsp90 and GSK3β, and incubation of the C-terminus of Hsp90α with PGK1 promoted the formation of the Hsp90-PGK1-GSK3β complex. These findings suggested that PGK1 was a recruiter co-chaperone that facilitated Hsp90 regulation of GSK3β stability. As a crucial ATP-generating enzyme in the glycolytic pathway, PGK1 catalyzes the dephosphorylation of 1,3-bisphosphoglycerate to 3-phosphoglycerate in the glycolysis pathway^30^. In our study, we identified PGK1 as a newly discovered recruiter co-chaperone that specially mediated Hsp90 regulation of GSK3β stability, which differed from previous findings that ATP generated from PGK1 may enhance the chaperone activity of Hsp90^28^. A large number of Hsp90 client proteins are closely related to cancer progression, drug-resistance, and maintenance of CSCs^48,49^. N-terminal inhibitors of Hsp90, including geldanamycin and 17-AAG, have been developed to antagonize cancer by binding to the ATP-binding sites of Hsp90 and thereby suppress its function^50^. HDN-1 has been identified as a C-terminal inhibitor^39^. It has been reported that Hsp90 clients have distinct sensitivities to different Hsp90 inhibitors^16^. In the present study, we showed that a decrease in GSK3β stability was induced by treatment with the Hsp90 inhibitor, 17-AAG, but not with HDN-1. Using 293T cells stably expressing the “closed” conformation of Hsp90α, we showed that the 17-AAG-induced decrease in GSK3β stability was due to its inhibitory effect on the interaction between Hsp90 and PGK1. These results indicated that HDN-1 and 17-AAG bound to Hsp90 in different conformational states, thereby exhibiting different effects on the interaction between Hsp90 and PGK1.

Most current clinical trials of Hsp90 inhibitors binding to the N-terminal ATP pocket of Hsp90 have been halted or postponed^15^. Owing to the inhibitory effects of Hsp90 on the stability of multiple proteins, toxic side effects and insufficient stratification of the patients could be possible reasons^15^. Detailed understanding of the specific recognition of Hsp90 with client proteins are therefore required for the development of new anticancer agents directed against Hsp90^51^. Here, we found that Hsp90 inhibitors targeting different sites had different effects on the interaction between Hsp90 and PGK1, which resulted in distinct effects on GSK3β stability, to affect the stemness of BCSCs, suggesting that Hsp90 inhibitors, which maintained GSK3β stability, could be effective in the treatment of breast cancer.

## Conclusions

We showed that GSK3β was a client protein of Hsp90 with PGK1 as a recruiter co-chaperone, which bound to the C-domain of the “closed” conformation of Hsp90. In addition, we also showed that Hsp90 inhibitors targeting different sites had distinct effects on the interaction between Hsp90 and PGK1. Our findings identified a novel regulatory mechanism of GSK3β stabilization with PGK1 as a co-chaperone of Hsp90, and emphasized the potential of selecting Hsp90 inhibitors with GSK3β stabilization for more effective cancer treatments.
