# Supplementary material for: PGK1-coupled HSP90 stabilizes GSK3β expression to regulate the stemness of breast cancer stem cells
Source: Cancer Biol Med. 2021 Aug 17;19(4):486–503. doi: 10.20892/j.issn.2095-3941.2020.0362 (PMC9088184; doi:10.20892/j.issn.2095-3941.2020.0362)
Supplement: Supplementary file 1 [file cbm-19-486-s001.pdf]

Supplementary materials

Table S1 The sequences of plasmid primers and siRNA

Plasmid/siRNA name	Primer/siRNA sequence
Flag-C-Hsp90α	
F1	5′-GGACGACGATGACAAGTTTGAGGGGAAGACTTTAGTGTC-3′
F2	5′-CCGCTCGAGATGGATTACAAGGACGACGATGACAAG-3′
R	5′-CGCGGATCCTTAGTCTACTTCTTCCATGCGTG-3′
Flag-N-Hsp90α	
F1	5′-GGACGACGATGACAAG GACCAACCGATGGAGGAGGAG-3′
F2	5′-CCGCTCGAGATGGATTACAAGGACGACGATGACAAG-3′
R	5′-CGCGGATCCTCATTCAGCCTCATCATCGCTTA-3′
Flag-M-Hsp90α	
F1	5′-GGACGACGATGACAAGAAGGAAGACAAAGAAGAAGAAAAAG-3′
F2	5′-CCGCTCGAGATG GATTACAAGGACGACGATGACAAG-3′
R	5′-CGCGGATCCTCACATATAGATCACTTCTAAGCC-3′
Flag-Hsp90α	
F	5′-CCGCTCGAGATGGATTACAAGGATGACGACGATAAGATGCCTGAGGAAACCCAGACC-3′
R	5′-CGCGGATCCTTAGTCTACTTCTTCCATGCGTG-3′
Flag-Hsp90α E47A	
F	5′-AAGAGATCTTTCTGAGAGCGCTCATTTCAAATTCATCA-3′
R	5′-TGATGAATTTGAAATGAGCGCTCTCAGAAAGATCTCTT-3′
Flag-Hsp90α D93A	
F	5′-GAACTCTCACTATTGTGGCTACTGGAATTGGAATGAC-3′
R	5′-GTCATTCCAATTCCAGTAGCCACAATAGTGAGAGTTC-3′
NC siRNA	Sense: 5′-UUCUCCGAACGUGUCACGUTT-3′;<br>Antisense: 5′-ACGUGACACGUUCGGAGAATT-3′.
Hsp90 siRNA	
#1	Sense: 5′-CCCUUCUAUUUGUCCCACGTT-3′;<br>Antisense: 5′-CGUGGGACAAAUAGAAGGGTT-3′.
#2	Sense: 5′-GGACAGUUGGAAUUCAGAGTT-3′;<br>Antisense: 5′-CUCUGAAUUCCAACUGUCCTT-3′.
PGK1 siRNA	
#1	Sense: 5′-GCUUCUGGGAACAAGGUUATT-3′;<br>Antisense: 5′-UAACCUUGUUCCCAGAAGCTT-3′.
#2	Sense: 5′-CCUGGAAGGUAAAGUCCUUTT-3′;<br>Antisense: 5′-AAGGACUUUACCUUCCAGGTT-3′.

Table S1 Continued

Plasmid/siRNA name	Primer/siRNA sequence
HOP siRNA	
#1	Sense: 5′-GCUACUCCGAAGCUAUUAATT-3′;<br>Antisense: 5′-UUAAUAGCUUCGGAGUAGCTT-3′.
#2	Sense: 5′-GCAAGACUGUCGACCUAAATT-3′;<br>Antisense: 5′-UUUAGGUCGACAGUCUUGCTT-3′.
#3	Sense: 5′-GCCAGAGCCAAUGGAAGAATT-3′;<br>Antisense: 5′-UUCUUCCAUUGGCUCUGGCTT-3′.
CDC37 siRNA	
#1	Sense: 5′-ACACAAGACCUUCGUGGAATT-3′;<br>Antisense: 5′-UUCCACGAAGGUCUUGUGUTT-3′.
#2	Sense: 5′-CGGCAGUUCUUCACUAAGATT-3′;<br>Antisense: 5′-UCUUAGUGAAGAACUGCCGTT-3′.
GSK3β siRNA	
#1	Sense: 5′-GCUGGAGUAUACACCAACUTT-3′;<br>Antisense: 5′-AGUUGGUGUAUACUCCAGCTT-3′.
#2	Sense: 5′-GGACAAGAGAUUUAAGAAUTT-3′;<br>Antisense: 5′-AUUCUUAAAUCUCUUGUCCTT-3′.

Table S2 Mass spectrometry analysis of proteins interacting with glycogen synthase kinase-3

Accession	Score	Mass	Matches	Sequences	emPAI	Name
sp|P35579|MYH9_HUMAN	1,026	227,646	47	32	0.84	Myosin-9 OS = Homo sapiens OX = 9606 GN = MYH9 PE = 1 SV = 4
sp|P60709|ACTB_HUMAN	344	42,052	18	10	2.11	Actin, cytoplasmic 1 OS = Homo sapiens OX = 9606 GN = ACTB PE = 1 SV = 1
tr|A0A075B6Z2|A0A075B6Z2_HUMAN	248	2,220	27	1	1.81	T cell receptor alpha joining 56 (Fragment) OS = Homo sapiens OX = 9606 GN = TRAJ56 PE = 4 SV = 1
tr|H0YH81|H0YH81_HUMAN	189	38,226	7	5	0.65	ATP synthase subunit beta (Fragment) OS = Homo sapiens OX = 9606 GN = ATP5F1B PE = 1 SV = 1
sp|P06702|S10A9_HUMAN	159	13,291	7	2	1.49	Protein S100-A9 OS = Homo sapiens OX = 9606 GN = S100A9 PE = 1 SV = 1
sp|Q02413|DSG1_HUMAN	139	114,702	7	7	0.22	Desmoglein-1 OS = Homo sapiens OX = 9606 GN = DSG1 PE = 1 SV = 2
sp|P31944|CASPE_HUMAN	138	27,947	5	5	0.76	Caspase-14 OS = Homo sapiens OX = 9606 GN = CASP14 PE = 1 SV = 2
sp|P81605|DCD_HUMAN	133	11,391	5	5	2.73	Dermcidin OS = Homo sapiens OX = 9606 GN = DCD PE = 1 SV = 2
tr|A0A0C4DGB6|A0A0C4DGB6_HUMAN	121	71,177	4	4	0.2	Serum albumin OS = Homo sapiens OX = 9606 GN = ALB PE = 1 SV = 1
tr|A0A087WVQ9|A0A087WVQ9_HUMAN	116	48,195	5	4	0.39	Elongation factor 1-alpha 1 OS = Homo sapiens OX = 9606 GN = EEF1A1 PE = 1 SV = 1
sp|Q6UWP8|SBSN_HUMAN	114	60,562	6	4	0.3	Suprabasin OS = Homo sapiens OX = 9606 GN = SBSN PE = 1 SV = 2
sp|P01040|CYTA_HUMAN	113	11,000	4	4	1.98	Cystatin-A OS = Homo sapiens OX = 9606 GN = CSTA PE = 1 SV = 1
sp|P14923|PLAK_HUMAN	109	82,434	5	4	0.22	Junction plakoglobin OS = Homo sapiens OX = 9606 GN = JUP PE = 1 SV = 3
sp|P00558|PGK1_HUMAN	99	44,985	5	3	0.43	Phosphoglycerate kinase 1 OS = Homo sapiens OX = 9606 GN = PGK1 PE = 1 SV = 3
sp|P15924|DESP_HUMAN	96	334,021	6	4	0.05	Desmoplakin OS = Homo sapiens OX = 9606 GN = DSP PE = 1 SV = 3
tr|F8VPF3|F8VPF3_HUMAN	95	14,598	3	2	0.87	Myosin light polypeptide 6 (Fragment) OS = Homo sapiens OX = 9606 GN = MYL6 PE = 1 SV = 1
sp|P12273|PIP_HUMAN	94	16,847	3	3	0.73	Prolactin-inducible protein OS = Homo sapiens OX = 9606 GN = PIP PE = 1 SV = 1
sp|Q9ULV4|COR1C_HUMAN	92	53,899	5	4	0.27	Coronin-1C OS = Homo sapiens OX = 9606 GN = CORO1C PE = 1 SV = 1
sp|P29508|SPB3_HUMAN	91	44,594	7	5	0.43	Serpin B3 OS = Homo sapiens OX = 9606 GN = SERPINB3 PE = 1 SV = 2
sp|P31151|S10A7_HUMAN	90	11,578	5	5	2.69	Protein S100-A7 OS = Homo sapiens OX = 9606 GN = S100A7 PE = 1 SV = 4
sp|P68871|HBB_HUMAN	90	16,102	3	3	0.77	Hemoglobin subunit beta OS = Homo sapiens OX = 9606 GN = HBB PE = 1 SV = 2
sp|P02489|CRYAA_HUMAN	86	20,011	3	1	0.17	Alpha-crystallin A chain OS = Homo sapiens OX = 9606 GN = CRYAA PE = 1 SV = 2
sp|Q5D862|FILA2_HUMAN	86	249,296	4	4	0.05	Filaggrin-2 OS = Homo sapiens OX = 9606 GN = FLG2 PE = 1 SV = 1
sp|Q71U36|TBA1A_HUMAN	82	50,788	4	3	0.21	Tubulin alpha-1A chain OS = Homo sapiens OX = 9606 GN = TUBA1A PE = 1 SV = 1
sp|P01834|IGKC_HUMAN	78	11,929	2	1	0.66	Immunoglobulin kappa constant OS = Homo sapiens OX = 9606 GN = IGKC PE = 1 SV = 2

Table S2 Continued

Accession	Score	Mass	Matches	Sequences	emPAI	Name
sp|P62805|H4_HUMAN	77	11,360	3	3	1.22	Histone H4 OS = Homo sapiens OX = 9606 GN = HIST1H4A PE = 1 SV = 2
sp|P07900|HS90A_HUMAN	77	85,006	1	1	0.04	Heat shock protein HSP 90-alpha OS = Homo sapiens OX = 9606 GN = HSP90AA1 PE = 1 SV = 5
tr|A0A0B4J259|A0A0B4J259_HUMAN	72	15,661	2	2	0.48	Lysozyme OS = Homo sapiens OX = 9606 GN = LYZ PE = 1 SV = 1
tr|J3QRS3|J3QRS3_HUMAN	70	20,501	2	2	0.35	Myosin regulatory light chain 12A OS = Homo sapiens OX = 9606 GN = MYL12A PE = 1 SV = 1
tr|Q5JP53|Q5JP53_HUMAN	66	48,135	3	3	0.22	Tubulin beta chain OS = Homo sapiens OX = 9606 GN = TUBB PE = 1 SV = 1
sp|Q96P63|SPB12_HUMAN	66	46,646	3	3	0.23	Serpin B12 OS = Homo sapiens OX = 9606 GN = SERPINB12 PE = 1 SV = 1
sp|Q08554|DSC1_HUMAN	66	101,406	4	2	0.1	Desmocollin-1 OS = Homo sapiens OX = 9606 GN = DSC1 PE = 1 SV = 2
sp|P05109|S10A8_HUMAN	65	10,885	4	3	2.01	Protein S100-A8 OS = Homo sapiens OX = 9606 GN = S100A8 PE = 1 SV = 1
sp|Q86YZ3|HORN_HUMAN	62	283,140	5	4	0.06	Hornerin OS = Homo sapiens OX = 9606 GN = HRNR PE = 1 SV = 2
tr|E7EUT5|E7EUT5_HUMAN	61	28,024	2	2	0.25	Glyceraldehyde-3-phosphate dehydrogenase OS = Homo sapiens OX = 9606 GN = GAPDH PE = 1 SV = 1
sp|P06733|ENOA_HUMAN	60	47,481	2	2	0.14	Alpha-enolase OS = Homo sapiens OX = 9606 GN = ENO1 PE = 1 SV = 2
sp|P07355|ANXA2_HUMAN	59	38,808	2	2	0.18	Annexin A2 OS = Homo sapiens OX = 9606 GN = ANXA2 PE = 1 SV = 2
sp|Q13835|PKP1_HUMAN	58	84,119	1	1	0.04	Plakophilin-1 OS = Homo sapiens OX = 9606 GN = PKP1 PE = 1 SV = 2
sp|P04040|CATA_HUMAN	56	59,947	2	2	0.11	Catalase OS = Homo sapiens OX = 9606 GN = CAT PE = 1 SV = 3
sp|Q08188|TGM3_HUMAN	56	76,926	3	3	0.13	Protein-glutamine gamma-glutamyltransferase E OS = Homo sapiens OX = 9606 GN = TGM3 PE = 1 SV = 4
tr|E9PKE3|E9PKE3_HUMAN	55	68,991	2	2	0.1	Heat shock cognate 71 kDa protein OS = Homo sapiens OX = 9606 GN = HSPA8 PE = 1 SV = 1
sp|Q9NZT1|CALL5_HUMAN	54	15,883	4	3	1.17	Calmodulin-like protein 5 OS = Homo sapiens OX = 9606 GN = CALML5 PE = 1 SV = 2
sp|P25705|ATPA_HUMAN	53	59,828	1	1	0.05	ATP synthase subunit alpha, mitochondrial OS = Homo sapiens OX = 9606 GN = ATP5F1A PE = 1 SV = 1
sp|P69905|HBA_HUMAN	53	15,305	1	1	0.22	Hemoglobin subunit alpha OS = Homo sapiens OX = 9606 GN = HBA1 PE = 1 SV = 2
sp|Q9HCY8|S10AE_HUMAN	52	11,826	1	1	0.29	Protein S100-A14 OS = Homo sapiens OX = 9606 GN = S100A14 PE = 1 SV = 1
sp|Q09666|AHNK_HUMAN	49	629,213	1	1	0.01	Neuroblast differentiation-associated protein AHNAK OS = Homo sapiens OX = 9606 GN = AHNAK PE = 1 SV = 2
sp|P10599|THIO_HUMAN	45	12,015	1	1	0.29	Thioredoxin OS = Homo sapiens OX = 9606 GN = TXN PE = 1 SV = 3
tr|A0A1B0GU92|A0A1B0GU92_HUMAN	43	60,677	1	1	0.05	Uncharacterized protein OS = Homo sapiens OX = 9606 PE = 1 SV = 1

Table S2 Continued

Accession	Score	Mass	Matches	Sequences	emPAI	Name
tr|B1AK87|B1AK87_HUMAN	41	29,562	1	1	0.11	Capping protein (actin filament) muscle Z-line, beta, isoform CRA_a OS = Homo sapiens OX = 9606 GN = CAPZB PE = 1 SV = 1
sp|P20930|FILA_HUMAN	41	435,036	2	2	0.02	Filaggrin OS = Homo sapiens OX = 9606 GN = FLG PE = 1 SV = 3
tr|A0A0U1RRH7|A0A0U1RRH7_HUMAN	39	18,541	1	1	0.18	Histone H2A OS = Homo sapiens OX = 9606 PE = 3 SV = 1
sp|Q01469|FABP5_HUMAN	38	15,497	1	1	0.22	Fatty acid-binding protein, epidermal OS = Homo sapiens OX = 9606 GN = FABP5 PE = 1 SV = 3
sp|P25311|ZA2G_HUMAN	37	34,465	2	2	0.2	Zinc-alpha-2-glycoprotein OS = Homo sapiens OX = 9606 GN = AZGP1 PE = 1 SV = 2
tr|F5GYA2|F5GYA2_HUMAN	37	28,749	2	1	0.12	Uracil-DNA glycosylase OS = Homo sapiens OX = 9606 GN = UNG PE = 1 SV = 1
sp|P47929|LEG7_HUMAN	37	15,123	1	1	0.23	Galectin-7 OS = Homo sapiens OX = 9606 GN = LGALS7 PE = 1 SV = 2
sp|P31025|LCN1_HUMAN	36	19,409	1	1	0.17	Lipocalin-1 OS = Homo sapiens OX = 9606 GN = LCN1 PE = 1 SV = 1
tr|H0YK49|H0YK49_HUMAN	35	24,259	2	1	0.29	Electron transfer flavoprotein subunit alpha, mitochondrial OS = Homo sapiens OX = 9606 GN = ETFA PE = 1 SV = 1
tr|A0A1W2PQ63|A0A1W2PQ63_HUMAN	34	84,612	1	1	0.04	Pleckstrin homology-like domain family B member 2 (Fragment) OS = Homo sapiens OX = 9606 GN = PHLDB2 PE = 1 SV = 1
sp|O75223|GGCT_HUMAN	32	21,222	2	2	0.34	Gamma-glutamylcyclotransferase OS = Homo sapiens OX = 9606 GN = GGCT PE = 1 SV = 1
tr|A0A087X0D5|A0A087X0D5_HUMAN	33	36,873	1	1	0.09	Pro-cathepsin H OS = Homo sapiens OX = 9606 GN = CTSH PE = 1 SV = 1
sp|P05089|ARGI1_HUMAN	32	34,884	3	2	0.2	Arginase-1 OS = Homo sapiens OX = 9606 GN = ARG1 PE = 1 SV = 2
sp|Q6R2W3|SCND3_HUMAN	32	153,452	1	1	0.02	SCAN domain-containing protein 3 OS = Homo sapiens OX = 9606 GN = ZBED9 PE = 2 SV = 1
sp|P06312|KV401_HUMAN	32	13,486	1	1	0.25	Immunoglobulin kappa variable 4-1 OS = Homo sapiens OX = 9606 GN = IGKV4-1 PE = 1 SV = 1
tr|M0R1V7|M0R1V7_HUMAN	32	7,128	2	2	1.25	Ubiquitin-60S ribosomal protein L40 (Fragment) OS = Homo sapiens OX = 9606 GN = UBA52 PE = 1 SV = 1
sp|O95969|SG1D2_HUMAN	31	10,260	1	1	0.34	Secretoglobin family 1D member 2 OS = Homo sapiens OX = 9606 GN = SCGB1D2 PE = 2 SV = 1
sp|Q8IW75|SPA12_HUMAN	31	47,259	2	2	0.14	Serpin A12 OS = Homo sapiens OX = 9606 GN = SERPINA12 PE = 1 SV = 1
sp|Q9NS67|GPR27_HUMAN	31	40,590	1	1	0.08	Probable G-protein coupled receptor 27 OS = Homo sapiens OX = 9606 GN = GPR27 PE = 2 SV = 1
sp|Q9H2G2|SLK_HUMAN	31	143,234	1	1	0.02	STE20-like serine/threonine-protein kinase OS = Homo sapiens OX = 9606 GN = SLK PE = 1 SV = 1
sp|P78524|ST5_HUMAN	31	127,603	1	1	0.03	Suppression of tumorigenicity 5 protein OS = Homo sapiens OX = 9606 GN = ST5 PE = 1 SV = 3

Table S2 Continued

Accession	Score	Mass	Matches	Sequences	emPAI	Name
sp|O94906|PRP6_HUMAN	30	107,656	1	1	0.03	Pre-mRNA-processing factor 6 OS = Homo sapiens OX = 9606 GN = PRPF6 PE = 1 SV = 1
sp|P53597|SUCA_HUMAN	30	36,626	1	1	0.09	Succinate--CoA ligase [ADP/GDP-forming] subunit alpha, mitochondrial OS = Homo sapiens OX = 9606 GN = SUCLG1 PE = 1 SV = 4
tr|E5RGS9|E5RGS9_HUMAN	30	6,260	1	1	0.58	Chromatin accessibility complex protein 1 OS = Homo sapiens OX = 9606 GN = CHRAC1 PE = 1 SV = 1
sp|Q8N436|CPXM2_HUMAN	30	86,443	1	1	0.04	Inactive carboxypeptidase-like protein X2 OS = Homo sapiens OX = 9606 GN = CPXM2 PE = 2 SV = 3
tr|F8WAJ0|F8WAJ0_HUMAN	29	84,148	1	1	0.04	RNA helicase OS = Homo sapiens OX = 9606 GN = DDX31 PE = 1 SV = 2
sp|Q9BPV8|P2Y13_HUMAN	29	41,275	1	1	0.08	P2Y purinoceptor 13 OS = Homo sapiens OX = 9606 GN = P2RY13 PE = 2 SV = 3
tr|K7EQQ1|K7EQQ1_HUMAN	28	18,732	1	1	0.18	Cleft lip and palate transmembrane protein 1 (Fragment) OS = Homo sapiens OX = 9606 GN = CLPTM1 PE = 1 SV = 1
tr|E7EQB2|E7EQB2_HUMAN	28	78,459	3	3	0.13	Lactotransferrin (Fragment) OS = Homo sapiens OX = 9606 GN = LTF PE = 1 SV = 1
tr|A0A0U1RQI5|A0A0U1RQI5_HUMAN	27	58,026	1	1	0.06	Sorbin and SH3 domain-containing protein 1 (Fragment) OS = Homo sapiens OX = 9606 GN = SORBS1 PE = 1 SV = 1
sp|P11279|LAMP1_HUMAN	27	45,367	1	1	0.07	Lysosome-associated membrane glycoprotein 1 OS = Homo sapiens OX = 9606 GN = LAMP1 PE = 1 SV = 3
tr|C9JM00|C9JM00_HUMAN	27	18,305	1	1	0.18	Serpin B7 (Fragment) OS = Homo sapiens OX = 9606 GN = SERPINB7 PE = 1 SV = 8
sp|P14618|KPYM_HUMAN	27	58,470	1	1	0.06	Pyruvate kinase PKM OS = Homo sapiens OX = 9606 GN = PKM PE = 1 SV = 4
tr|A0A087WX19|A0A087WX19_HUMAN	26	76,884	1	1	0.04	IQ domain-containing protein E OS = Homo sapiens OX = 9606 GN = IQCE PE = 1 SV = 1
tr|R4GMN1|R4GMN1_HUMAN	26	56,043	1	1	0.06	Motile sperm domain-containing protein 2 OS = Homo sapiens OX = 9606 GN = MOSPD2 PE = 1 SV = 1
tr|A0A286YES1|A0A286YES1_HUMAN	26	50,242	1	1	0.07	Immunoglobulin heavy constant gamma 3 (Fragment) OS = Homo sapiens OX = 9606 GN = IGHG3 PE = 1 SV = 1
sp|Q9ULJ3|ZBT21_HUMAN	26	120,564	1	1	0.03	Zinc finger and BTB domain-containing protein 21 OS = Homo sapiens OX = 9606 GN = ZBTB21 PE = 1 SV = 2
sp|O94776|MTA2_HUMAN	25	75,717	1	1	0.04	Metastasis-associated protein MTA2 OS = Homo sapiens OX = 9606 GN = MTA2 PE = 1 SV = 1
sp|Q7Z2Z1|TICRR_HUMAN	25	212,721	1	1	0.02	Treslin OS = Homo sapiens OX = 9606 GN = TICRR PE = 1 SV = 2
sp|P22528|SPR1B_HUMAN	25	10,337	1	1	0.34	Cornifin-B OS = Homo sapiens OX = 9606 GN = SPRR1B PE = 1 SV = 2
sp|Q5T750|XP32_HUMAN	25	28,557	1	1	0.12	Skin-specific protein 32 OS = Homo sapiens OX = 9606 GN = XP32 PE = 1 SV = 1
tr|K7EL99|K7EL99_HUMAN	24	14,836	1	1	0.23	Tubulin-folding cofactor B (Fragment) OS = Homo sapiens OX = 9606 GN = TBCB PE = 1 SV = 2

Table S2 Continued

Accession	Score	Mass	Matches	Sequences	emPAI	Name
tr|H7C4W4|H7C4W4_HUMAN	23	2,420	1	1	1.61	Follistatin-related protein 1 (Fragment) OS = Homo sapiens OX = 9606 GN = FSTL1 PE = 1 SV = 1
tr|A0A0G2JQD0|A0A0G2JQD0_HUMAN	23	63,847	1	1	0.05	Lanosterol synthase (Fragment) OS = Homo sapiens OX = 9606 GN = LSS PE = 1 SV = 1
sp|Q14807|KIF22_HUMAN	23	73,616	1	1	0.04	Kinesin-like protein KIF22 OS = Homo sapiens OX = 9606 GN = KIF22 PE = 1 SV = 5
sp|Q9BX63|FANCJ_HUMAN	23	142,785	2	1	0.02	Fanconi anemia group J protein OS = Homo sapiens OX = 9606 GN = BRIP1 PE = 1 SV = 1
sp|Q92556|ELMO1_HUMAN	23	84,517	1	1	0.04	Engulfment and cell motility protein 1 OS = Homo sapiens OX = 9606 GN = ELMO1 PE = 1 SV = 2
tr|F5H070|F5H070_HUMAN	23	150,248	1	1	0.02	Lysine-specific demethylase 3A OS = Homo sapiens OX = 9606 GN = KDM3A PE = 1 SV = 2
sp|O94988|FA13A_HUMAN	23	117,600	1	1	0.03	Protein FAM13A OS = Homo sapiens OX = 9606 GN = FAM13A PE = 1 SV = 2
tr|H3BN02|H3BN02_HUMAN	23	129,653	1	1	0.03	Integrin alpha-X OS = Homo sapiens OX = 9606 GN = ITGAX PE = 1 SV = 1
tr|H0YE71|H0YE71_HUMAN	23	10,673	1	1	0.32	Uncharacterized protein OBSCN-AS1 (Fragment) OS = Homo sapiens OX = 9606 GN = OBSCN-AS1 PE = 4 SV = 1
tr|F5H6E2|F5H6E2_HUMAN	22	119,719	1	1	0.03	Unconventional myosin-Ic OS = Homo sapiens OX = 9606 GN = MYO1C PE = 1 SV = 1
tr|A0A0D9SEN1|A0A0D9SEN1_HUMAN	21	88,213	1	1	0.04	Prolyl endopeptidase FAP OS = Homo sapiens OX = 9606 GN = FAP PE = 1 SV = 1
sp|Q8NBJ4|GOLM1_HUMAN	21	45,477	1	1	0.07	Golgi membrane protein 1 OS = Homo sapiens OX = 9606 GN = GOLM1 PE = 1 SV = 1
tr|U3KPT7|U3KPT7_HUMAN	20	25,117	1	1	0.13	Synaptosomal-associated protein 47 (Fragment) OS = Homo sapiens OX = 9606 GN = SNAP47 PE = 1 SV = 1
sp|Q9NV70|EXOC1_HUMAN	20	102,772	1	1	0.03	Exocyst complex component 1 OS = Homo sapiens OX = 9606 GN = EXOC1 PE = 1 SV = 4
sp|Q6NWY9|PR40B_HUMAN	19	99,638	1	1	0.03	Pre-mRNA-processing factor 40 homolog B OS = Homo sapiens OX = 9606 GN = PRPF40B PE = 1 SV = 1
sp|Q9H2Y7|ZN106_HUMAN	19	210,693	1	1	0.02	Zinc finger protein 106 OS = Homo sapiens OX = 9606 GN = ZNF106 PE = 1 SV = 1
tr|E5RIG8|E5RIG8_HUMAN	19	15,899	1	1	0.21	Pro-neuregulin-1, membrane-bound isoform (Fragment) OS = Homo sapiens OX = 9606 GN = NRG1 PE = 1 SV = 1
sp|Q92540|SMG7_HUMAN	19	127,886	1	1	0.03	Protein SMG7 OS = Homo sapiens OX = 9606 GN = SMG7 PE = 1 SV = 2
tr|E7ERF2|E7ERF2_HUMAN	19	47,394	1	1	0.07	T-complex protein 1 subunit alpha OS = Homo sapiens OX = 9606 GN = TCP1 PE = 1 SV = 1
tr|A0A0G2JMZ6|A0A0G2JMZ6_HUMAN	18	99,019	1	1	0.03	Kinesin-like protein (Fragment) OS = Homo sapiens OX = 9606 GN = KIF5C PE = 1 SV = 1
tr|M0QZJ5|M0QZJ5_HUMAN	17	25,892	1	1	0.13	Calcium-binding mitochondrial carrier protein SCaMC-3 (Fragment) OS = Homo sapiens OX = 9606 GN = SLC25A23 PE = 1 SV = 1

Table S2 Continued

Accession	Score	Mass	Matches	Sequences	emPAI	Name
tr|H0Y798|H0Y798_HUMAN	16	9,189	1	1	0.38	Propionyl-CoA carboxylase alpha chain, mitochondrial (Fragment) OS = Homo sapiens OX = 9606 GN = PCCA PE = 1 SV = 1
tr|H0YIP0|H0YIP0_HUMAN	16	24,350	1	1	0.14	Methionine--tRNA ligase, cytoplasmic (Fragment) OS = Homo sapiens OX = 9606 GN = MARS PE = 1 SV = 1
sp|Q4VNC0|AT135_HUMAN	16	139,119	1	1	0.02	Probable cation-transporting ATPase 13A5 OS = Homo sapiens OX = 9606 GN = ATP13A5 PE = 2 SV = 1
sp|Q6PIF6|MYO7B_HUMAN	16	243,612	1	1	0.01	Unconventional myosin-VIIb OS = Homo sapiens OX = 9606 GN = MYO7B PE = 1 SV = 2
tr|H0Y525|H0Y525_HUMAN	15	8,648	1	1	0.4	Ethylmalonyl-CoA decarboxylase (Fragment) OS = Homo sapiens OX = 9606 GN = ECHDC1 PE = 1 SV = 1
sp|Q6E0U4|DMKN_HUMAN	15	47,282	1	1	0.07	Dermokine OS = Homo sapiens OX = 9606 GN = DMKN PE = 1 SV = 3
